# Non-invasive and objective tear film breakup detection on interference color images using convolutional neural networks

**DOI:** 10.1371/journal.pone.0282973

**Published:** 2023-03-13

**Authors:** Yasushi Kikukawa, Shin Tanaka, Takuya Kosugi, Stephen C. Pflugfelder

**Affiliations:** 1 Kowa Ophthalmic Research Laboratories, Kowa Research Institute, Inc., Boston, Massachusetts, United States of America; 2 Kowa Company, Ltd., Tokyo, Japan; 3 Department of Ophthalmology, Baylor College of Medicine, Houston, Texas, United States of America; King Faisal University, SAUDI ARABIA

## Abstract

**Purpose:**

Dry eye disease affects hundreds of millions of people worldwide and is one of the most common causes for visits to eye care practitioners. The fluorescein tear breakup time test is currently widely used to diagnose dry eye disease, but it is an invasive and subjective method, thus resulting in variability in diagnostic results. This study aimed to develop an objective method to detect tear breakup using the convolutional neural networks on the tear film images taken by the non-invasive device KOWA DR-1α.

**Methods:**

The image classification models for detecting characteristics of tear film images were constructed using transfer learning of the pre-trained ResNet50 model. The models were trained using a total of 9,089 image patches extracted from video data of 350 eyes of 178 subjects taken by the KOWA DR-1α. The trained models were evaluated based on the classification results for each class and overall accuracy of the test data in the six-fold cross validation. The performance of the tear breakup detection method using the models was evaluated by calculating the area under curve (AUC) of receiver operating characteristic, sensitivity, and specificity using the detection results of 13,471 frame images with breakup presence/absence labels.

**Results:**

The performance of the trained models was 92.3%, 83.4%, and 95.2% for accuracy, sensitivity, and specificity, respectively in classifying the test data into the tear breakup or non-breakup group. Our method using the trained models achieved an AUC of 0.898, a sensitivity of 84.3%, and a specificity of 83.3% in detecting tear breakup for a frame image.

**Conclusions:**

We were able to develop a method to detect tear breakup on images taken by the KOWA DR-1α. This method could be applied to the clinical use of non-invasive and objective tear breakup time test.

## Introduction

Dry eye disease (DED) affects hundreds of millions of people worldwide and is one of the most common causes of visits to eye care practitioners. It is defined as a multifactorial disease of the ocular surface characterized by a loss of homeostasis of the tear film, and accompanied by ocular symptoms, in which tear film instability and hyperosmolarity, ocular surface inflammation and damage, and neurosensory abnormalities play etiological roles [1].

Age, gender, computer use, and contact lens wear are considered risk factors for DED. The prevalence of DED varies depending on the condition and region of the study, but it is generally high, with a reported prevalence of 6.8% in adults of the United States, affecting approximately 16.4 million people [2, 3]. The effects of DED, such as reduced vision, quality of life, and work productivity, are considered to be an economic burden to society.

It is important to evaluate the stability of the tear film for DED diagnosis, and the fluorescein breakup time (FBUT) test has been widely used in clinical practice [4–6]. FBUT is measured as the time elapsed between a complete blink and the appearance of the first breakup in the tear film after sodium fluorescein is instilled into the test eye [7]. When performing FBUT instillation of fluorescein dye has been found to decrease the stability of the tear film [8, 9]. The quantity and concentration of fluorescein instilled during the test can also affect the FBUT measurement [7]. In addition, since the test is basically a manual and subjective measurement, it is difficult to obtain reproducible results and there is a tendency for inter-examiner variability. There are two issues with the FBUT test: it is subjective and invasive.

In recent years, there has been a lot of research on the use of artificial intelligence (AI) in the medical field, and an automated AI diagnostic system for diabetic retinopathy was approved by the United States Food and Drug Administration (USFDA) in 2018 [10]. Among AI technologies, convolutional neural networks (CNNs), which are one of the deep learning methods, have been attracting attention in image classification and identification, and many studies using CNNs have been reported in the field of eye care [11–14]. Su et al. developed an automatic method to measure FBUT using a CNN model that learned visual features of fluorescein stained images of the ocular surface to detect tear breakup [15].

The Tear Film and Ocular Surface Society (TFOS) international Dry Eye Workshop II (DEWS II) report recommends the measurement of non-invasive tear breakup time (NIBUT) as the test for tear film stability [7, 16]. There are several commercially available non-invasive test devices based on topographic or videokeratographic methods. These devices measure NIBUT by analyzing changes in the reflected placido mires projected on the ocular surface [7, 17].

**Fig 1:**
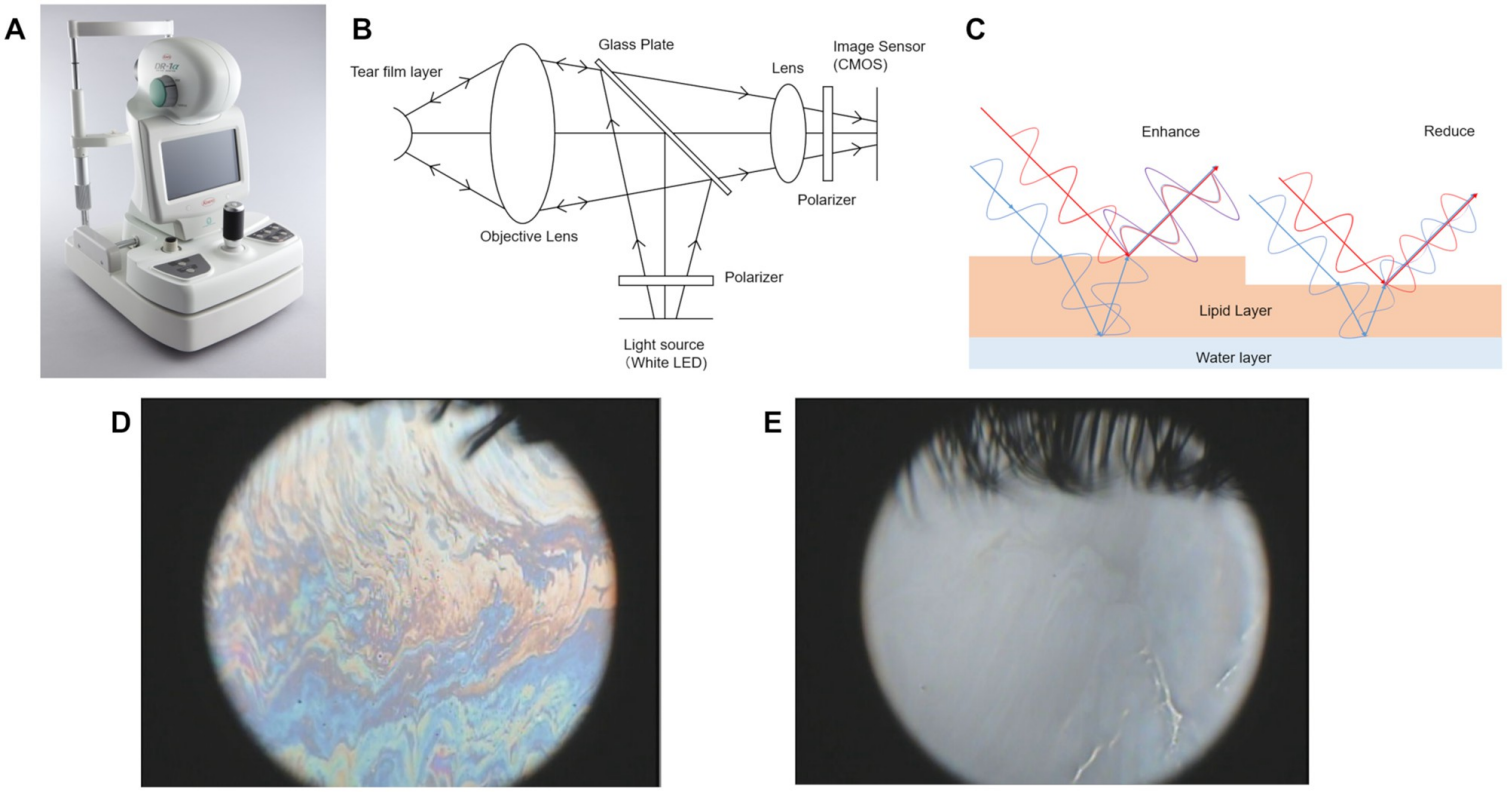
Overview of the KOWA DR-1α. (A) Appearance of the device. (B) Optical path diagram. (C) Principle of interference. (D) Example of interference color image. (E) Example of interference color image with tear breakup.

The KOWA DR-1α video interferometer (Kowa Company, Ltd., Tokyo, Japan) is a device that allows non-invasive observation of the tear film dynamics of the entire cornea by projecting white light onto the tear film and using the optical interference color image created by the difference between the light reflected from the front surface of the lipid layer and the light reflected from the back surface (Fig 1) [18]. Since the KOWA DR-1α enables to observe tear breakup patterns equivalent to those observed with fluorescein staining from the images taken, it is possible to measure the TBUT subjectively by visual inspection [19]. The NIBUT measurement of commercially available devices detect tear breakup indirectly, whereas the KOWA DR-1α can directly observe tear breakup. Various studies have been conducted on the KOWA DR-1α, including tear lipid layer grading system, measurement of lipid layer thickness (LLT), measurement of tear meniscus height (TMH), and classification of dry eye subtypes, but no study has been reported on objective and automatic detection of tear breakup yet [7, 17, 18]. Several elements such as interference fringes and oil particles appear in the tear interference color images, which may require training and experience to subjectively detect tear breakup by visual inspection. Therefore, an image classification model that identifies the characteristics of tear interference color images was constructed using CNN.

We have developed a method to detect tear breakup on the tear film images taken by the KOWA DR-1α using our image classification model toward realizing a non-invasive and objective tear film stability test.

## Materials and methods

### Data collection

Retrospective review and analysis of study data was approved by the Institutional Review Board of Baylor College of Medicine (IRB No. H-51925). The need for informed consent was waived by the IRB because of the retrospective design of the study. It adhered to the tenets of the Declaration of Helsinki for clinical research.

The examinations for this study were conducted at Alkek Eye Center (Houston, Texas, USA) from September 30, 2019 to March 8, 2021. The exam was performed on both dry eye patients and healthy subjects and a total of 183 participants were included in the study. The diagnostic criteria for DED were a Symptom Assessment in Dry Eye (SANDE) score of >80 and a FBUT of <10 seconds. We used 350 eye data (female 272 and male 78, and dry eye 303 and healthy 47) from 178 participants (mean age 59.98±15.09 years, female 138 and male 40, and dry eye 153 and healthy 25), excluding 5 participants for insufficient data.

The KOWA DR-1α test was performed according to the NIBUT measurement on the DEWS II report, with the instructions to blink naturally three times and then keep the eye open as long as possible [7]. The tear interference color video was taken for 30 seconds by the KOWA DR-1α’s built-in camera. The video was recorded with a resolution of 640 x 480 pixels at 30 frames per second.

### Construction and evaluation of CNN model

We used the ResNet50 model which was pre-trained on the ImageNet dataset to build a CNN model for detecting characteristics of tear interference color images by performing transfer learning on images extracted from videos recorded by the KOWA DR-1α [20–22]. The ResNet50 model consisted of 49 convolutional layers and one fully-connected layer, and had over 23 million trainable parameters, so that this model architecture demonstrated successful performance when applied to image classification [20]. The model used in this study was pre-trained on the ImageNet including about 1,000 categories, and was a model from the machine learning library Keras [21]. For transfer learning, the final fully-connected layer was removed, the other layers were frozen and adopted as fixed feature extractor, and the new fully-connected layer was trained with the dataset prepared in this study using the softmax activation function so that the output was nine classes described below.

The image classification performed by our CNN model was defined to classify into nine classes, three breakup related classes and six non-breakup related classes by analyzing the characteristic elements in the tear interference color images. Although the shape and size of tear breakup vary from case to case, the image size to perform the image classification was defined as 96 x 96 pixels so that each class can be distinguished. The training data used to train the CNN model was created by using our dedicated software to select any frame image of the video and extracting a 96x96 pixels image patch of the area where the desired classification is displayed.

**Fig 2:**
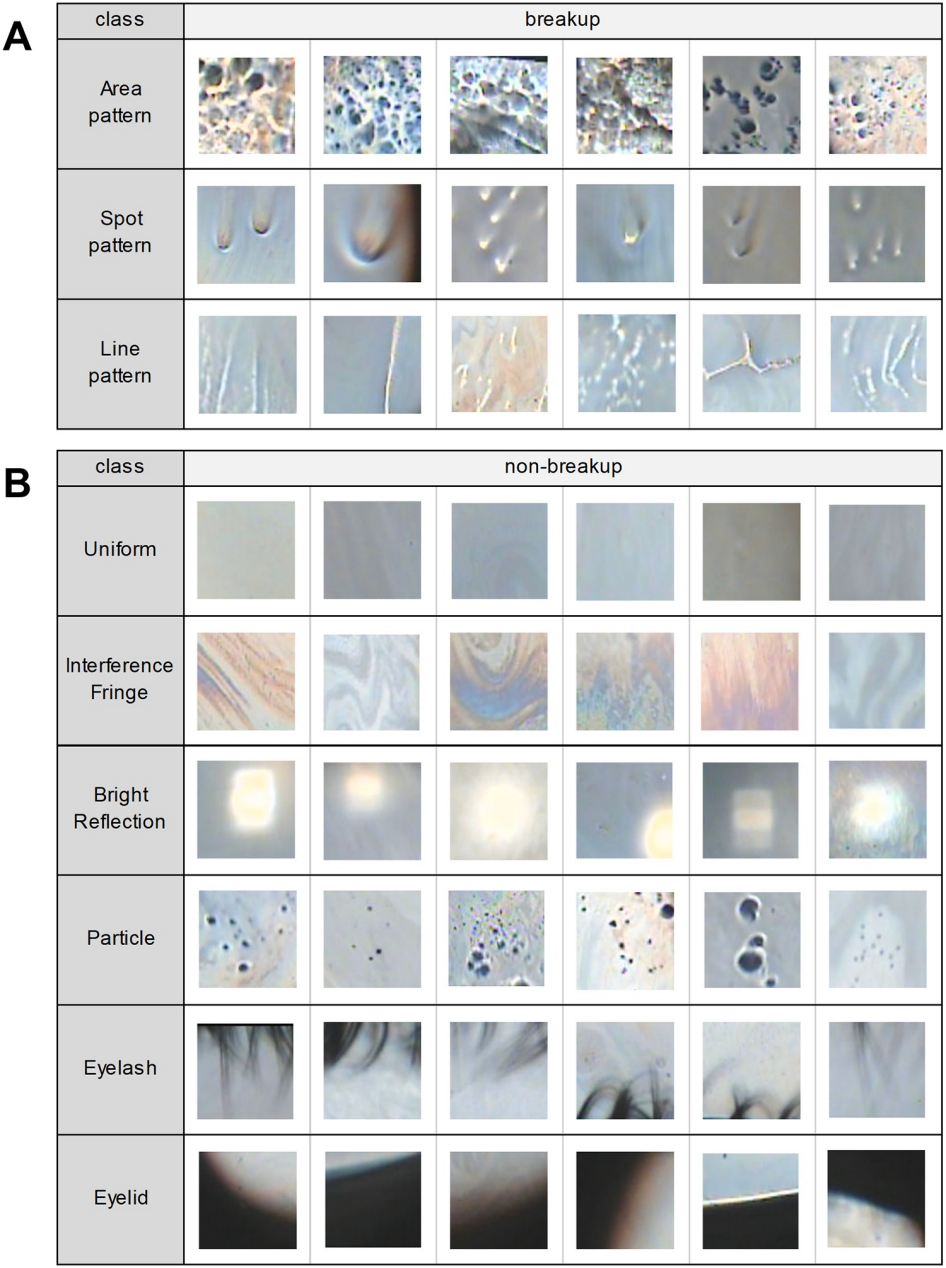
Examples of image patches for each class of training data. (A) Tear breakup classes. (B) Non-breakup classes.

The breakup category was classified into three classes: Area pattern, Spot pattern, and Line pattern. In studies of tear breakup patterns (BUPs) observed by fluorescein staining, various BUPs have been reported based on the timing of occurrence, location and shape of tear breakup [23], but when BUPs are classified by shape, they can be summarized into three types. Therefore, since the purpose of this study is to detect tear breakup, we classified the tear breakup category into three classes. The Area pattern class was defined as an image with tear breakup where the corneal surface was appeared to be exposed due to thinning of the tear film. The Spot pattern class was defined as an image with circular breakup, and the Line pattern class was defined as an image with linear breakup. Example image patches of each tear breakup class are shown in Fig 2A. In addition, examples of the three tear breakup types observed in the tear interference color images taken with the KOWA DR-1α are shown in S1 Fig.

The non-breakup category was classified into six classes: Uniform, Interference Fringe, Bright Reflection, Particle, Eyelash, and Eyelid. The Uniform class was defined as an interference image of the tear film in which the interference color was one color and there was no stripe pattern or it was too thin to be seen clearly, and the Interference Fringe class was defined as an interference image in which the interference stripe pattern could be seen clearly. The Bright Reflection class was defined as an image containing areas of higher brightness than the surrounding area, as seen in eyes with intraocular lenses, and the Particle class was defined as an image containing black objects such as oil particles or debris. The Eyelash class was defined as an image containing the upper or lower eyelashes, and the Eyelid class was defined as an image containing a part of the upper or lower eyelids or the circular camera mask. Example image patches of each non-breakup class are shown in Fig 2B.

Strong interference fringes were sometimes difficult to distinguish from the Line pattern, so the Interference Fringe class was established as one independent class in our image classification. In addition, the strong reflections observed in eyes with intraocular lenses could be misclassified as the Spot pattern, so the Bright Reflection class was established as an independent class.

The training data was created by three engineers who had previously trained the tear breakup detection criteria with a specialist. A total of 9,089 image data of nine classes was prepared as shown in Table 1.

**Table 1:** Number of images for each class of training data.

category	breakup			non-breakup						total
class	Area pattern	Spot pattern	Line pattern	Uniform	Interference Fringe	Bright Reflection	Particle	Eyelash	Eyelid	
**number of images**	643	684	877	1099	1201	1046	1230	1119	1190	9089

The training of our CNN model was performed in a six-fold cross-validation. We divided the training data into six groups so that images extracted from the same video belonged to the same group, and that there was no class bias among the groups (Tables 2 and 3).

**Table 2:** Detailed breakdown of the six groups of training data.

Group	breakup			non-breakup						number of eyes
	Area pattern	Spot pattern	Line pattern	Uniform	Interference Fringe	Bright Reflection	Particle	Eyelash	Eyelid	
**A**	107	131	184	174	203	183	207	202	203	60
**B**	106	106	135	203	194	188	208	296	180	56
**C**	111	118	150	177	206	164	207	207	204	57
**D**	100	112	131	167	192	149	207	261	205	60
**E**	109	107	134	174	202	163	196	177	200	59
**F**	110	110	143	204	204	199	205	176	198	58
**total**	643	684	877	1099	1201	1046	1230	1319	1190	350

**Table 3:** Combination of groups for six-fold cross-validation.

No.	Learning					Test
	Training				Validation	
**1**	A	B	C	D	E	F
**2**	F	A	B	C	D	E
**3**	E	F	A	B	C	D
**4**	D	E	F	A	B	C
**5**	C	D	E	F	A	B
**6**	B	C	D	E	F	A

As a preprocessing for training, data augmentation was performed to increase the diversity of the training data [24]. Specifically, scaling, horizontal and vertical shifting, and horizontal flipping were applied to the images of the training data. Vertical flipping was not applied because some of the breakup patterns have vertical shape characteristics. The images of the training data were rescaled to a resolution 224 x 224 pixels to match the input size of the CNN model. The training process was performed as follows. The optimization function was adam, the error function was categorical crossentropy, the activation function of the output layer was softmax, the batch size was 20, and the number of cycles was 1,000 epochs. The model when the accuracy of the validation data was maximized in the training process was adopted as the training result. The training process was conducted using two workstation PCs, one with an NVIDIA Quadro P200 GPU, and the other with an NVIDIA Quadro P2200 GPU. The ResNet50 model used in this study was implemented using Keras version 2.0.8 and Tensorflow-1.10.0-gpu. Our training program was written in the Python programming language (Python 3.5 Python Software Foundation).

The trained models were evaluated for image classification performance using the results of the test data in the six-fold cross validation. Accuracy, recall, precision, and F1-score were used as performance metrics.

### Method and evaluation of tear breakup detection using CNN model

**Fig 3:**
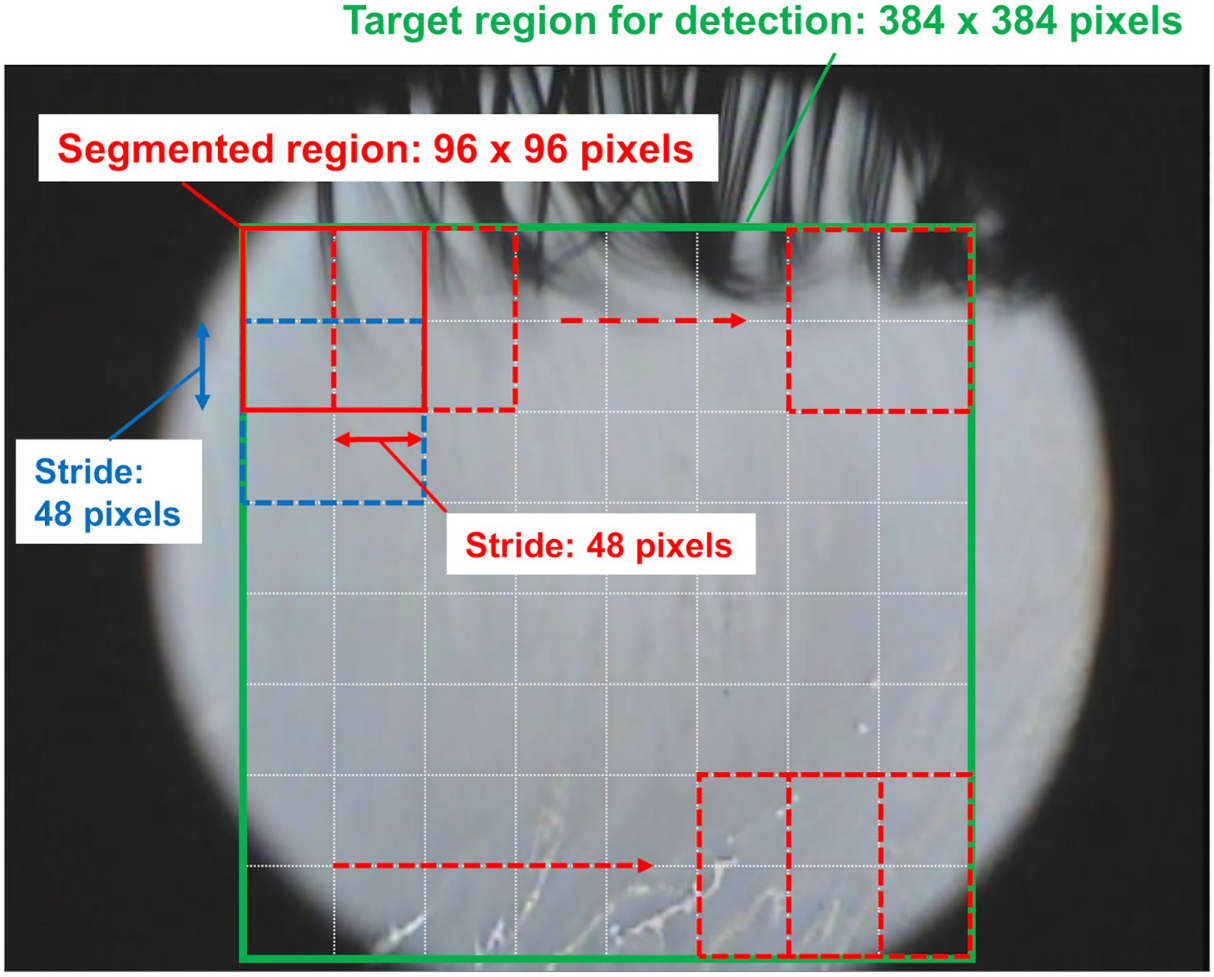
Method for tear breakup detection in a frame image. The target region for detection (384 x 384 pixels) is divided into 96 x 96 pixels segments with a stride length of 48 pixels, and each segment region is classified by the trained model. When the number of segments classified as breakup is equal to or greater than a certain number, the frame image is determined to have tear breakup.

The detection procedure for tear breakup on a frame image of the KOWA DR-1α video using the trained CNN model was as follows. A 384 x 384 pixels region near the center of a frame image, where almost the entire cornea was captured, was used for detection. The target region was divided into 96 x 96 pixels segmented regions with a stride length of 48 pixels. Adjacent segmented regions were overlapped by half a region to prevent missing detection (Fig 3). Each segmented region was classified by our CNN model. If the number of segmented regions classified as breakup (Area, Spot, or Line pattern) was equal to or greater than a certain number, the frame image was determined to have tear breakup.

We prepared 13,471 frame images labeled with the presence or absence of tear breakup from 350 eye videos to evaluate our detection method. The frame images for evaluation were created by extracting up to 40 frame images from a single video and labeling the extracted frame images with the presence or absence of tear breakup using our dedicated software. The work was carried out by three engineers who were trained in the tear breakup detection criteria.

The program for the detection procedure was written in Python 3.5. The detection process of the prepared frame images was conducted on a workstation PC with an NVIDIA Quadro P2000 GPU. A receiver operating characteristic (ROC) analysis was performed to evaluate the detection method. The performance of the method was expressed as the area under curve (AUC) of ROC curve with 95% confidence interval (95% CI), sensitivity, and specificity. The statistical programming language R (Version 4.0.3, The R Foundation for Statistical Computing) was used for the statistical analysis.

## Results

### Image classification results of CNN models

**Fig 4:**
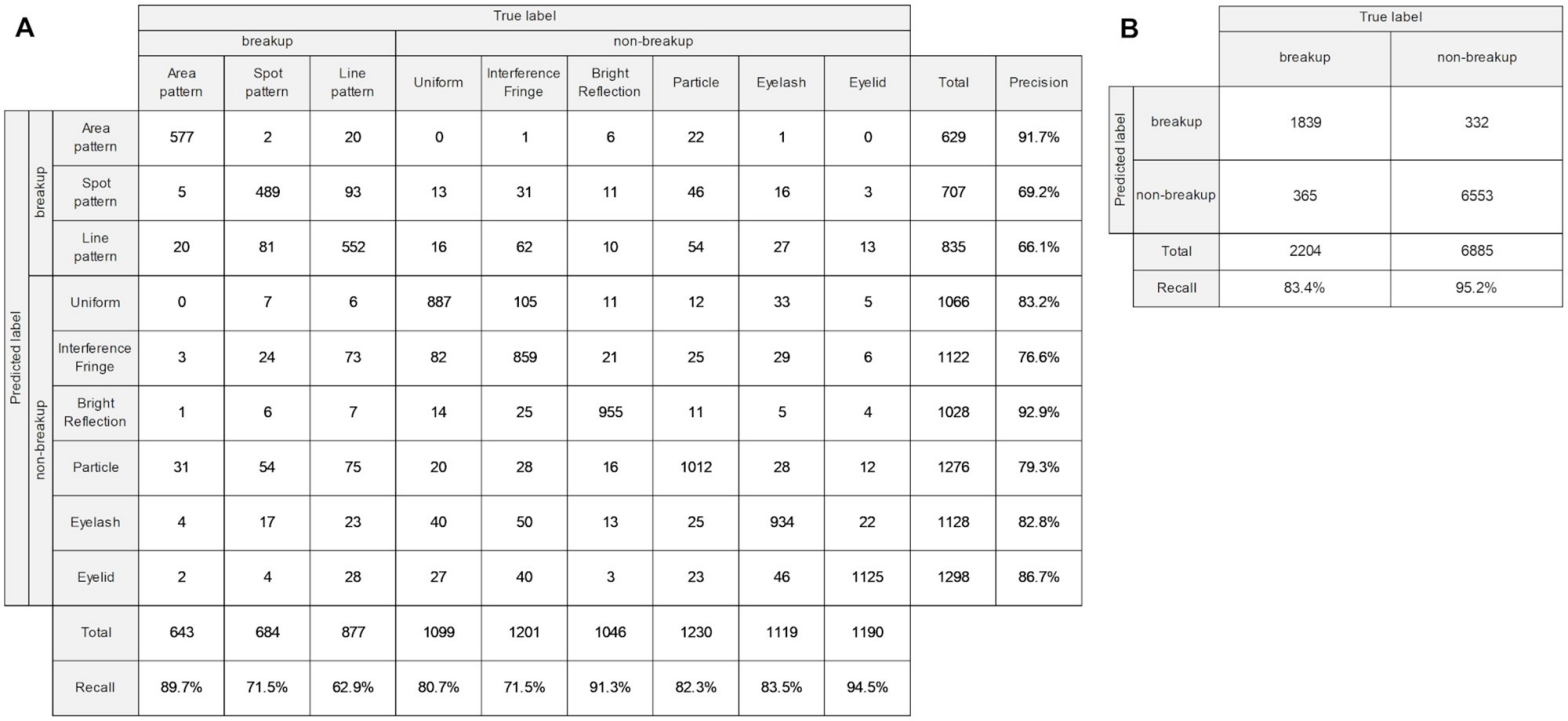
Confusion matrices. (A) Confusion matrix for image classification of test data in six-fold cross validation. (B) Confusion matrix aggregated for breakup and non-breakup.

The computation time (mean ± standard deviation) per epoch for the training process was 109.30±9.88 seconds with an NVIDIA Quadro P2000 GPU and 81.50±1.45 seconds with an NVIDIA Quadro P2200 GPU. The test results of the six CNN models trained by six-fold cross validation are shown in the confusion matrix in Fig 4A. The overall accuracy was 81.3%. The recalls of Area pattern, Spot pattern, and Line pattern related to breakup category were 89.7%, 71.5%, and 62.9%, respectively. The recalls of Uniform, Interference Fringe, Bright Reflection, Particle, Eyelash, and Eyelid related to non-breakup category were 80.7%, 71.5%, 91.3%, 82.3%, 83.5%, and 94.5%, respectively. The precisions of Area pattern, Spot pattern, and Line pattern were 91.7%, 69.2%, and 66.1%, respectively. The precisions of Uniform, Interference Fringe, Bright Reflection, Particle, Eyelash, and Eyelid were 83.2%, 76.6%, 92.9%, 79.3%82.8%, and 86.7%, respectively. The F1-scores of Area pattern, Spot pattern, and Line pattern were 0.907, 0.703, and 0.645, respectively. The F1-scores of Uniform, Interference Fringe, Bright Reflection, Particle, Eyelash, and Eyelid were 0.819, 0.740, 0.921, 0.808, 0.831, and 0.904, respectively. The results of six-fold cross-validation, expressed as mean ± standard deviation, were 81.3%±2.53, 81.0%±2.24, 81.5%±2.49, and 0.810±0.022 for accuracy, recall, precision, and F1-score, respectively.

Fig 4B shows the confusion matrix aggregated into two groups: breakup and non-breakup. The ability of our CNN model to classify into the tear breakup or non-breakup group was 92.3%, 83.4%, and 95.2% for accuracy, sensitivity, and specificity, respectively. The results of six-fold cross-validation, expressed as mean ± standard deviation, were 92.3%±0.91, 83.7%±5.44, and 95.1%±2.80 for accuracy, sensitivity, and specificity, respectively.

### Evaluation results of tear breakup detection method

The detection process of 13,471 frame images took 4 hours and 5 minutes. Thus, the computation time per frame image was approximately 1.1 seconds.

**Fig 5:**
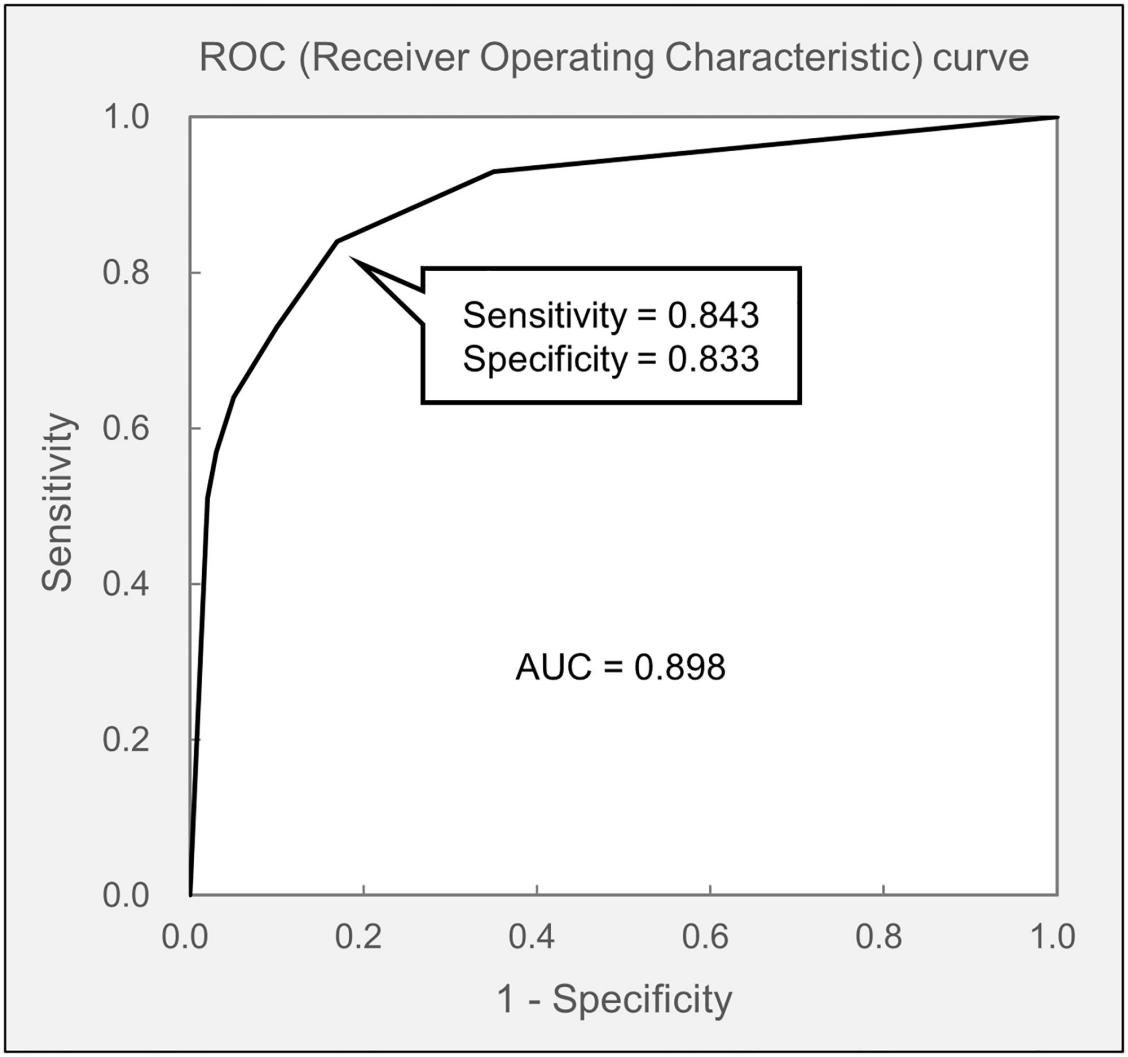
Receiver operating characteristic (ROC) curve. This curve shows that our method achieved an area under curve (AUC) of 0.898, a sensitivity of 84.3%, and a specificity of 83.3% in detecting tear breakup for a frame image.

The evaluation results of the method for detecting tear breakup for a frame image are shown in the receiver operating characteristic (ROC) curve in Fig 5. Our method achieved to detect tear breakup with sensitivity and specificity of 84.3% and 83.3%, respectively. The area under the curve (AUC) was 0.898 (95% CI: 0.891 to 0.905).

## Discussion

**Fig 6:**
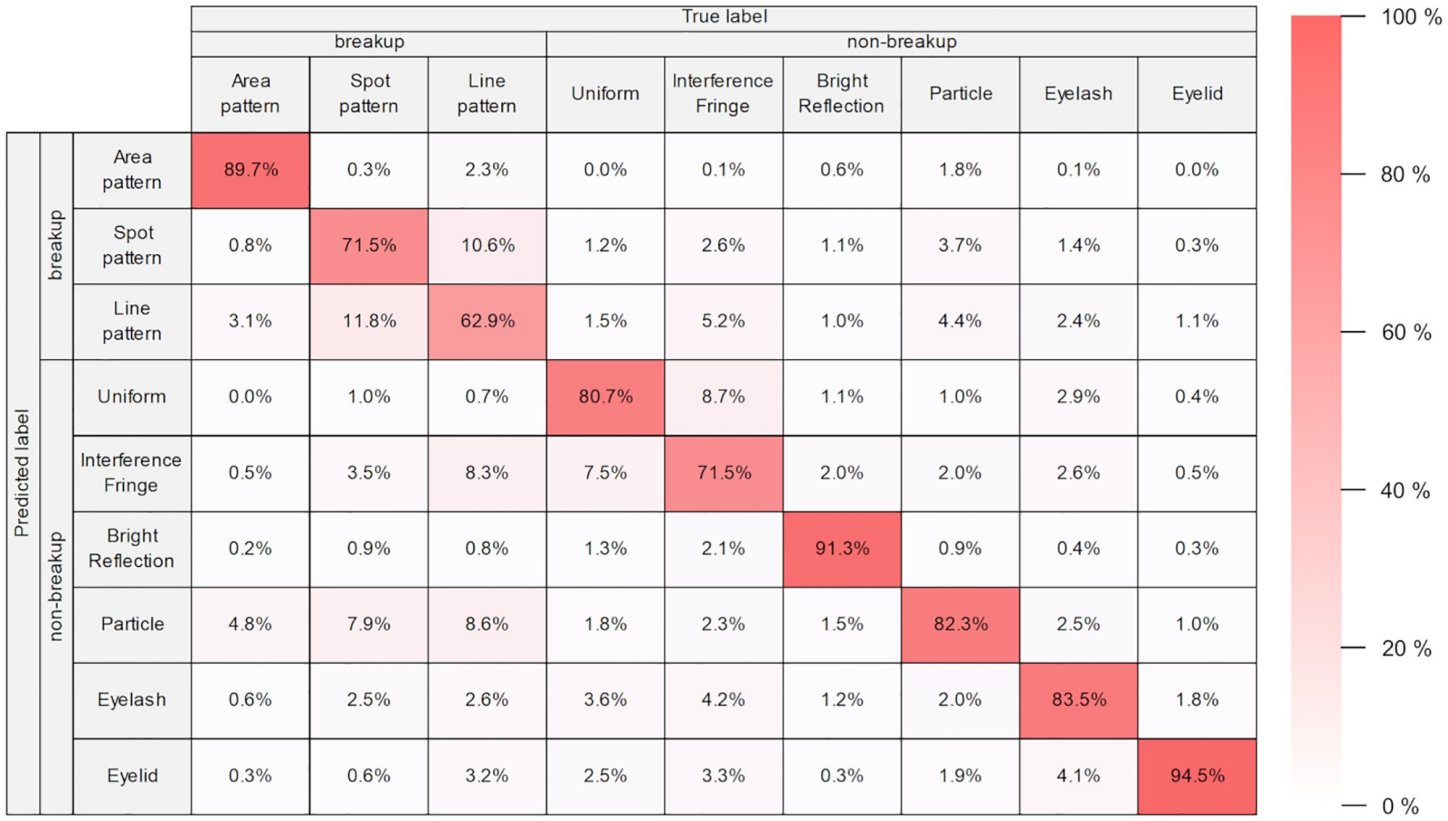
Heatmap confusion matrix. Each cell shows the ratio of the number of predicted data to the number of actual data.

**Fig 7:**
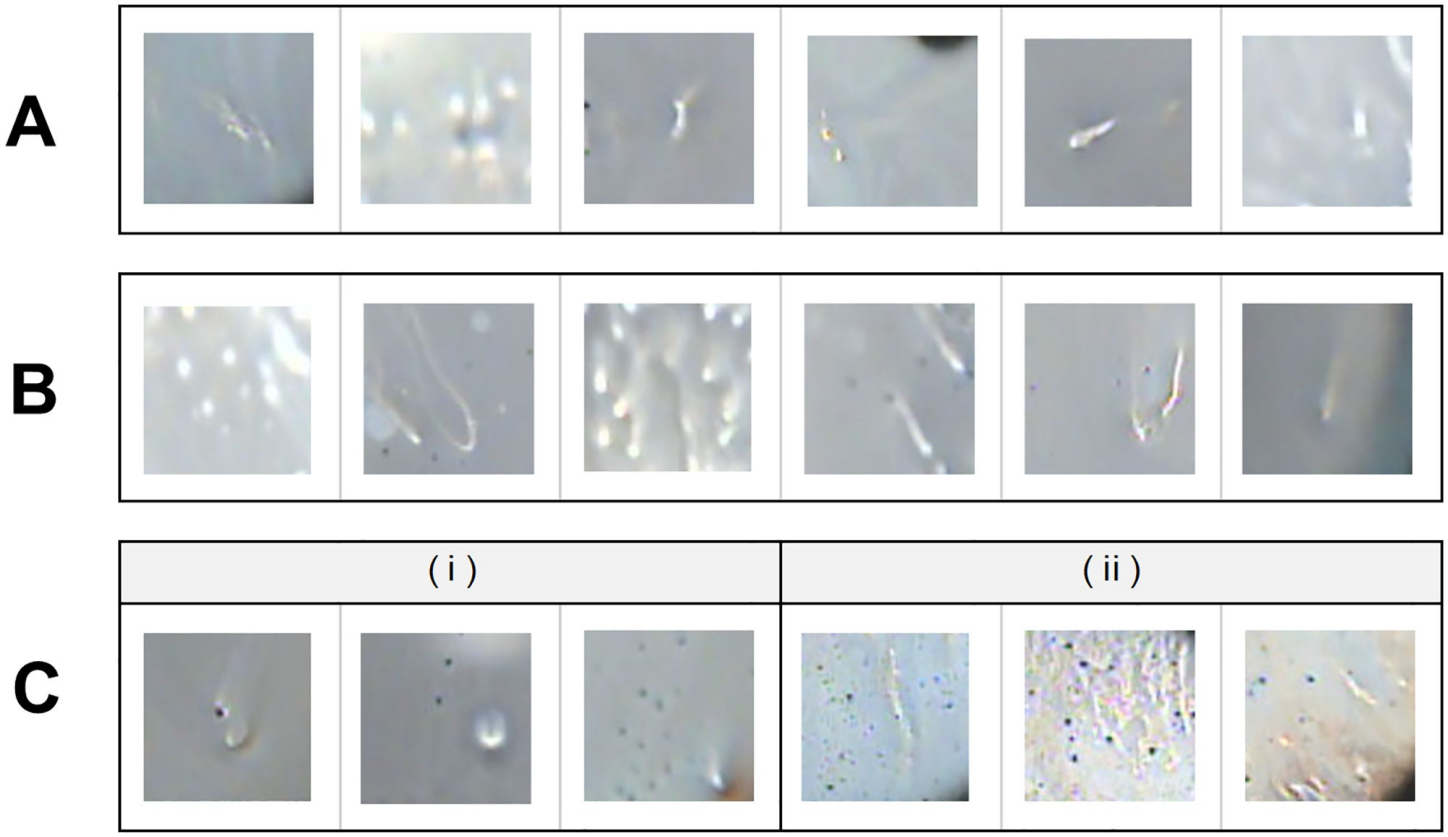
Examples of misclassified images. (A) Line pattern misclassified to Spot pattern. (B) Spot pattern misclassified to Line pattern. (C) Breakup patterns misclassified to Particle: (i) Spot pattern and (ii) Line pattern.

To discuss the details of the image classification performance of the trained CNN models, the confusion matrix which is expressed as the ratio of the number of predicted data to the number of actual data is shown in Fig 6. The trained CNN models were able to classify Area pattern, Bright Reflection, and Eyelid with high recalls of approximately 90% or better. Uniform, Particle, and Eyelash were also classified with more than 80% recalls. On the other hand, the recalls for Spot pattern, Line pattern, and Interference Fringe were lower than those for the other classes. The Spot pattern and Line pattern tended to be misclassified into each other. This misclassification may be due to the following reasons. The Line pattern at the time of occurrence could be similar in shape to the Spot pattern due to its shorter length (Fig 7A). Also, the Spot pattern at a few seconds after occurrence could be similar in shape to the Line pattern due to the shape change caused by the upward movement of tear fluid (Fig 7B).

The Spot pattern and Line pattern tended to be misclassified as the Particle class. When there were the Spot or Line pattern breakup and black particles in the same region, our CNN model sometimes classified them into the Particle class (Fig 7C).

In order to reduce the misclassification of the Line pattern breakup and interference fringes, the Interference Fringe class was established and trained, but there was still a tendency for them to misclassify each other. In general, the performance of AI depends on the quality and quantity of the training data, so if we can use a lot of good data for training, we can improve the accuracy of our CNN model further.

**Fig 8:**
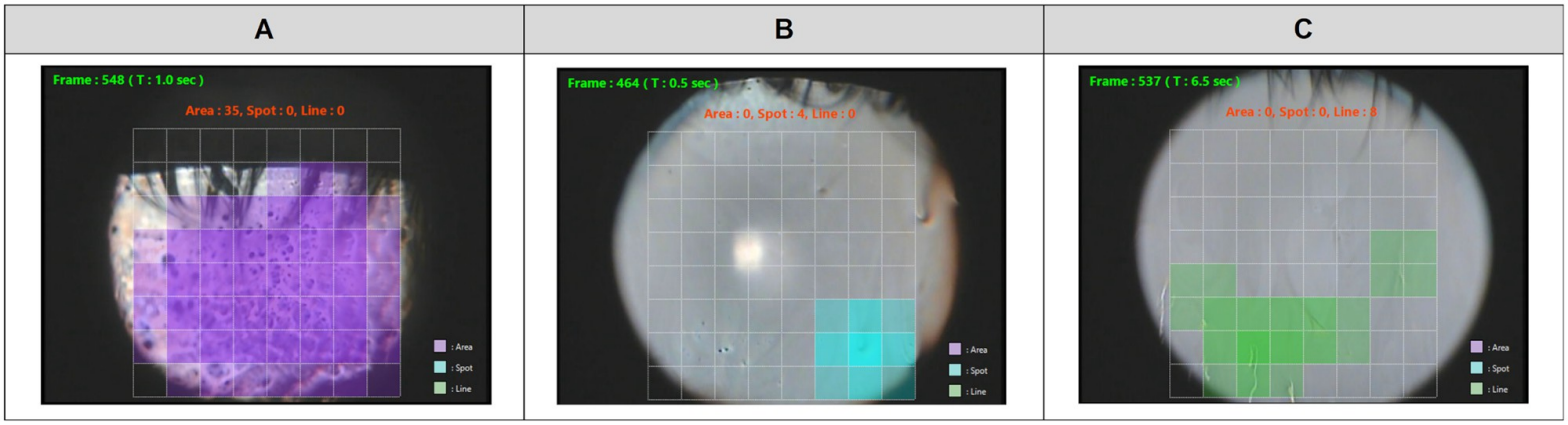
Examples of a frame image where breakup was detected correctly. The colored regions are where tear breakup was detected: (A) Area pattern (purple), (B) Spot pattern (blue), and (C) Line pattern (green).

Since the method developed in this study for detecting tear breakup for a frame image was based on the results of image classification of the CNN model, the trend of the results basically matched the trend of the results of image classification of the CNN model. The detection of tear breakup in a frame image had a sensitivity of 84.3% and a specificity of 83.3%, and the presence or absence of tear breakup occurrence could be determined with satisfactory accuracy (Fig 8).

**Fig 9:**
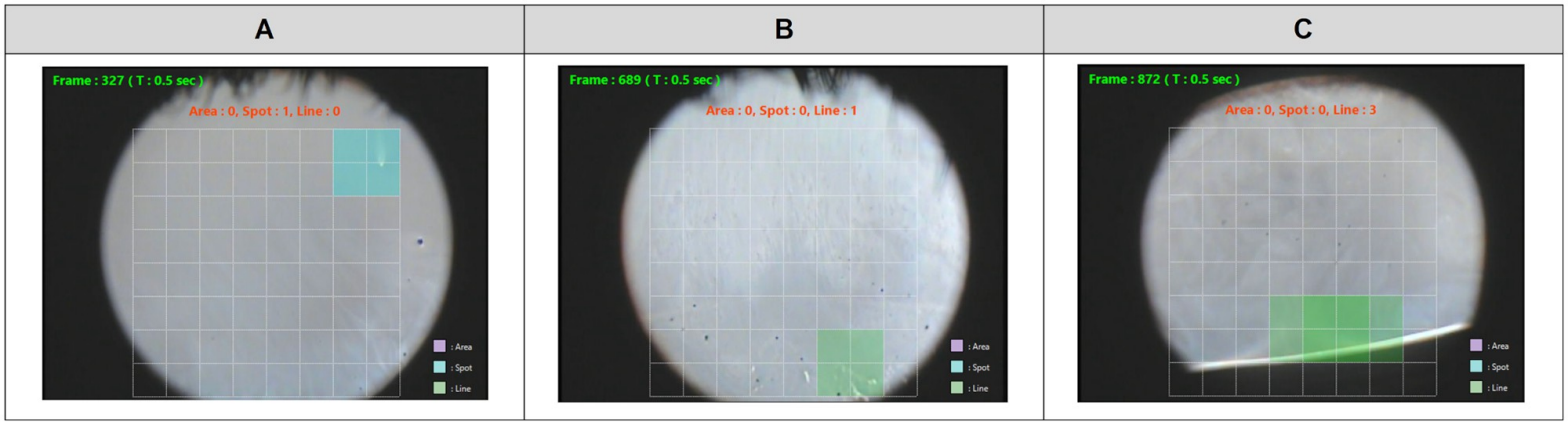
Examples of a frame image where detection of breakup failed. In (A) and (B), the breakup detection area was too small to determine the presence of breakup: (A) Spot pattern and (B) Line pattern. In (C), the high intensity reflections along the lower eyelid were incorrectly detected as Line pattern breakup.

Considering the cases where the detection of tear breakup failed, we found that it was sometimes determined that no tear breakup occurred when the area of tear breakup was small (Fig 9). Therefore, it may be possible that tear breakup can be detected by optimizing the conditions for judging the occurrence of tear breakup depending on the type of breakup pattern.

High intensity reflections observed along the edge of the eyelid were sometimes mistakenly detected as the Line pattern class (Fig 9). Also, blurred tear breakup, which is difficult to determine even for experts, was sometimes classified as a non-breakup class. These are expected to be improved by improving the accuracy of the CNN model.

## Conclusions

We were able to develop a method for detecting tear breakup by using a CNN model trained on the characteristics of tear film interference color images of the KOWA DR-1α video interferometer. This method could be practical for non-invasive and objective TBUT measurement, which would be useful for dry eye diagnosis in clinical practice.

## Supporting information

S1 Fig Examples of three tear breakup types observed in tear interference color images taken with the KOWA DR-1α. (A) Area pattern. (B) Spot pattern. (C) Line pattern.
(TIF)
