# Supplementary figures and images for: Non-invasive and objective tear film breakup detection on interference color images using convolutional neural networks
Source: PLoS One. 2023 Mar 13;18(3):e0282973. doi: 10.1371/journal.pone.0282973 (PMC10010540; doi:10.1371/journal.pone.0282973)

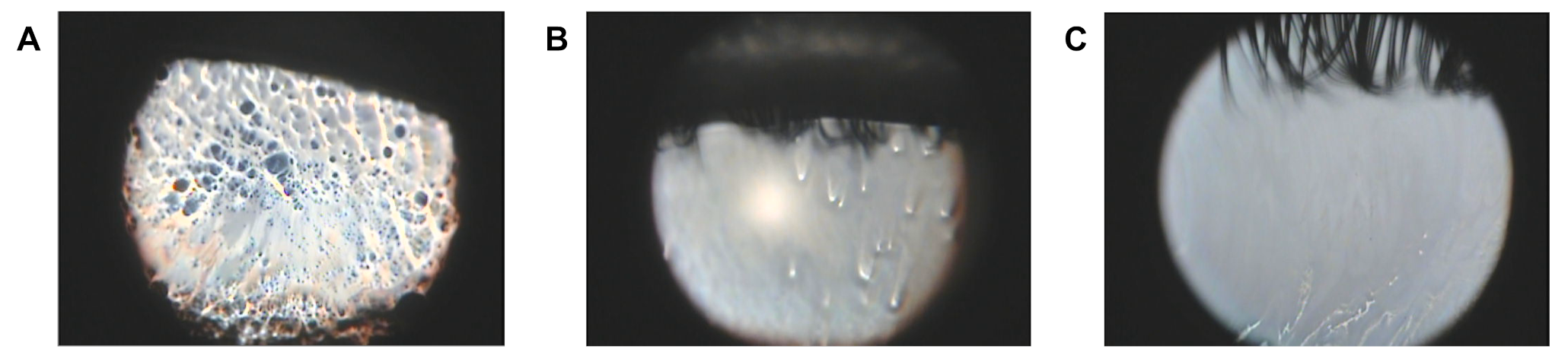

Supplement: S1 Fig — (A) Area pattern. (B) Spot pattern. (C) Line pattern. (TIF) [file pone.0282973.s001.tif]
